# Reference intervals for serum TSH concentrations of healthy children from the Central Region of Brazil

**DOI:** 10.20945/2359-4292-2022-0499

**Published:** 2023-06-19

**Authors:** Tula Beatriz Brandão Caldas Meirelles-Cardoso, Natasha Slhessarenko, Cor Jesus Fernandes Fontes

**Affiliations:** 1 Universidade Federal de Mato Grosso Hospital Universitário Júlio Müller Departamento de Clínica Médica Cuiabá MT Brasil Hospital Universitário Júlio Müller, Universidade Federal de Mato Grosso, Departamento de Clínica Médica, Cuiabá, MT, Brasil; 2 Universidade de Cuiabá Faculdade de Medicina Cuiabá MT Brasil Universidade de Cuiabá, Faculdade de Medicina, Cuiabá, MT, Brasil; 3 Universidade Federal de Mato Grosso Faculdade de Medicina Departamento de Pediatria Cuiabá MT Brasil Faculdade de Medicina, Universidade Federal de Mato Grosso, Departamento de Pediatria, Cuiabá, MT, Brasil; 4 Alta Excelência Diagnóstica São Paulo SP Brasil Alta Excelência Diagnóstica (DASA), São Paulo, SP, Brasil; 5 Centro Universitário de Várzea Grande Várzea Grande MT Brasil Centro Universitário de Várzea Grande (UNIVAG), Várzea Grande, MT, Brasil

**Keywords:** Reference intervals, thyroid-stimulating hormone, healthy children, Brazil

## Abstract

**Objective:**

The objective of this study was to determine the serum thyroid-stimulating hormone (TSH) concentration reference intervals (RIs) of healthy children aged 1 to 10 years of both sexes, living in the Central Region of Brazil.

**Subjects and methods:**

1,735 children [869 (50.1%) female; 866 (49.9%) male] enrolled in the morning shift of 47 pre- and 83 public elementary schools in the municipality of Cuiabá, Mato Grosso, were studied by gathering anthropometric and social data and their medical history. A blood sample was collected from each child to determine the TSH concentration using the electrochemiluminescence method on a Cobas® 6000 modular analyzer (Analyzer series, Roche Diagnostics).

**Results:**

The RIs were determined using the 2.5 and 97.5 percentile and the mean ± 2 standard deviations methods. After identifying the homoscedastic groups by age and sex, outliers higher or lower than three standard deviations were excluded. The distribution of serum TSH concentrations showed no significant age or sex differences. Based on the percentile method, TSH RI ranged from 0.93 to 5.86 μIU/mL. Based on the mean ± 2 standard deviations, TSH RI ranged from 0.30 to 5.29 μIU/mL.

**Conclusion:**

The normal serum TSH concentration of the Brazilian children evaluated in this study differ from those of populations from other countries. Other regional population studies may validate the RIs found in this study and enable its safer use in pediatric clinical practice.

## INTRODUCTION

Clinical laboratory tests play a key role in the diagnosis, monitoring and treatment of various diseases. However, the interpretation of results from these tests is often limited by the lack of appropriate reference intervals (RIs) for local populations (1–4).

In pediatric clinical practice, establishing RIs is particularly challenging given the continuous physiological changes that occur during childhood. Establishing accurate pediatric RIs of a healthy population is crucial for correctly interpretating clinical laboratory results, particularly for immunochemical analytes (5,6). Direct and appropriately selected sampling from a reference population is the approach recommended by the Clinical and Laboratory Standards Institute (CLSI). In addition, assessing and subsequently excluding any unhealthy individual present in the sample increases the validity and adequacy of RIs (7). Therefore, reference individuals should be as similar as possible to the future target patient, in all respects except for the disease or condition under study (8).

In thyroid disorders, correctly interpreting test results is essential to their diagnosis, because thyroid disease signs and symptoms are subtle or absent at disease onset in most patients. Only sensitive and specific biochemical tests can detect these disorders, as long as they are specifically validated for the target population (8).

Effective thyroid function is crucial for growth and development in young people. Even small variations in thyroid hormone homeostasis are associated with growth and energy expenditure sequelae and with metabolic disturbances (9). Measuring serum TSH concentrations is the most reliable test for diagnosing primary hypothyroidism and hyperthyroidism, especially in outpatients (10,11).

Elevated concentrations close to the upper threshold of normal serum TSH concentration may translate into the diagnosis of subclinical hypothyroidism (SH). Generally, TSH concentrations above the upper limit of the reference interval, in the presence of normal concentrations of free thyroxine (FT4), defines clinical hypothyroidism (CH) (12). However, the normal serum TSH concentration varies among populations (7,8) and there is still no consensus on the serum TSH concentration cutoff point for SH diagnosis (13). Previous studies have suggested lowering the upper limit of normal TSH in adults to define SH. However, for practical purposes, and with potential therapeutic implications, this evidence was not justified (14). Among children, this evidence was not justified given the variety of TSH RIs across study groups on this subject in this population (15–17).

In Brazil, serum TSH concentration RIs for children are based on results from international studies or from internal studies of the medical device industry, often conducted with laboratory, outpatient or hospital samples, which can affect the concentration of the hormone (18). The thyroid analysis consensuses of the Brazilian Society of Endocrinology and Metabolism (*Sociedade Brasileira de Endocrinologia e Metabologia* – SBEM) are also based on different international studies (8,19). In addition, the heterogeneity in device, determination method and children's age range of available studies hinders the interpretation of different RIs (17,20–23). Given the need to standardize a laboratory test to diagnose thyroid dysfunction in the Brazilian pediatric population, the present study was designed to establish the serum TSH concentration RI of healthy children aged from 1 to 10 years of both sexes of a population living in a municipality of the Central Region of Brazil. This study hypothesizes that the normal serum TSH concentrations in the healthy Brazilian pediatric population differ from those observed in children from other countries or regions worldwide, for genetic, cultural or environmental reasons.

## SUBJECTS AND METHODS

This observational, cross-sectional, descriptive study was conducted with children from the city of Cuiabá, Mato Grosso, located in the Central-West Region of Brazil. The population of this city primarily consists of migrants from various regions of the country, who were motivated by a government campaign aimed at dispersing the Brazilian population to sparsely populated areas, during the 1970s (24,25).

This research was performed with secondary data from a study conducted in 2012 to determine the RIs of various serum analyte concentrations of healthy children and adolescents of the municipality of Cuiabá, Mato Grosso, Brazil (26). The sample size was calculated from the mean values and their expected variability in the population for all analytes under study, using data from American studies as reference (5), assuming a normal distribution of their values in the population. Based on these criteria, the sample size was calculated for the analyte with the largest sample. Children without any known underlying disease, clinical signs or symptoms, and health-related complaints at the time of blood sample collection were included in the study.

The following parameters were analyzed to calculate the sample size: i) the expected standard deviation of serum TSH concentration in the population was 2.0 μIU/mL (27); ii) Student's t-distribution to estimate the sample size in a finite population of 70,000 children enrolled in pre- and elementary schools in the city of Cuiabá (28); iii) 2.0 design effect of the cluster sampling procedure; iv) 5% alpha error and 80% power. With these assumptions, a sample of 2,260 children aged from 2 to 10 years was defined for the study.

All pre- and elementary schools of the municipality of Cuiabá, distributed in the North, South, East and West regions of the municipality, were included in the study. In the first stage of the cluster sampling procedure, five pre- and five elementary schools were randomly selected by region of the city. In the second stage, the children were randomly selected for analysis. Of the 2,260 children predicted in the sampling procedure, 1,994 healthy children aged from one to 10 years, attending the morning shift of 20 pre- and 25 public elementary schools in the city, were included in this study. This sample size makes it possible to estimate the mean TSH serum concentration in the population, with 95% confidence and ± 0.1 μIU/mL accuracy (29).

Legal guardians of all children were interviewed to gather their demographic and clinical data and medical history. A blood sample was collected by venipuncture from a superficial forearm vein, according to CLSI guidelines (30), using small gauge needles. Since the original study measured multiple analytes, a three-hour fast was recommended for children aged from one to two years; a six-hour fast, for children aged two to five years; and a 12-hour fast, for children older than five years. The determination of serum TSH concentration was processed on the same day as blood collection.

The children's weight was measured using a digital scale, and height was measured using an infantometer when the children were younger than two years, or a stadiometer, when the children were two years of age or older. Birth weight data were gathered from the children's legal guardians and classified as low birth weight when lower than 2,500 g, normal weight when ranging from 2,500 to 4,000 g, and macrosomia when higher than 4,000 g (31). The children's nutritional status was determined by age z-score based on the body mass index, using the software WHO AnthroPlus, version 3.2.2 (32).

All blood samples were processed in the Cobas® 6000 modular analyzer (Analyzer series-Roche Diagnostics, Indianapolis, USA) on the same day of collection and analyzed using the electrochemiluminescence methodology. Automatic methods provided by all the major diagnostic companies are extremely sensitive and reproducible, and are widely available for clinical use (Thienpont and cols., 2010). TSH measurements using automatic methods of Roche are very similar to the post-harmonization standard values recommended by the IFCC C-STFT, even at low concentration ranges (33,34). The serum TSH concentration was measured *at Diagnósticos da América S/A* laboratory in Cuiabá, which is International Organization for Standardization (ISO) 9001-certified for clinical laboratory quality and good practices. This certification was issued by the Brazilian Society of Clinical Pathology/Laboratory Medicine (*Sociedade Brasileira de Patologia Clínica/Medicina Laboratorial* – SBPC/ML). Data were entered into an Microsoft Excel spreadsheet and analyzed using the Stata statistical package, version 12.0 (Stata Corp, Texas, USA).

Initially, the Shapiro-Wilk test was performed to assess the distribution of serum TSH concentrations and their variance between groups of children, by sex and age. Because the distribution of these concentrations throughout the study sample was asymmetric, the non-parametric Kruskal-Wallis test was performed to define sex and age groups, that is, groups with homogeneous variances of serum TSH concentrations between sexes and between the ten age groups from 1 to 10 years.

Once homoscedastic groups were defined by sex and age, the serum TSH concentrations statistically summarized to identify outliers in these groups. Any value exceeding the mean ± 3.0 standard deviations was considered an outlier and was, therefore, excluded from the analysis. Once the outliers were excluded, serum TSH serum RIs were set using the 2.5 and 97.5 percentile and mean ± 2 standard deviation methods (3,8).

As part of the results of the initial study were used in a doctoral thesis presented to the Graduate Program in Pediatrics at USP, this study was approved by the Faculty of Medicine of the University of São Paulo (*Faculdade de Medicina da Universidade de São Paulo* – FMUSP; 318/2011) and Júlio Müller University Hospital (*Hospital Universitário Júlio Müller* – HUJM; 947/2010) Research Ethics Committees. The present analysis was also approved by the HUJM Research Ethics Committee (Certificate of Presentation for Ethical Consideration (*Certificado de Apresentação para Apreciação Ética* – CAAE: 45600921.4.0000.5541 of 04/27/2021).

## RESULTS

Of the 1,994 children recruited for this study, 131 were excluded because they were 10 years of age by the assessment date, 114 because they had symptoms at the time of blood collection, and 14 because they were regularly using medication for some clinical condition. Therefore, 1,735 healthy children participated in this study. Their father and/or mother were/was invited to visit the pre- or elementary school for an interview and asked to sign the informed consent form for their child's participation in this study. During the interview, the guardians failed to provide data on the requested characteristics of sociodemographic, perinatal, and anthropometric data, either for overlooking or forgetting them. For this reason, the sample number varied in some analyses.

The mean ± standard deviation (SD) of the participating children's age was 5.3 ± 2.7 years, with 50.1% girls and 49.9% boys. Based on the self-reported skin color of 1,725 (99.4%) children, 51 (3.0%) were yellow; 215 (12.5%), black; 343 (19.9%), white; and 1,116 (64.7%), brown. The mode of delivery was reported by 1,729 (99.6%) mothers of these children, of whom 915 (52.9%) were born by vaginal delivery and 814 (47.1%) by cesarean delivery. Among children with information about breastfeeding in the first year of life, most (82.8%) were breastfed for 4.7 months on average. The mean ± SD birth weight reported for the participating children was 3,226 ± 577 grams (Table 1).

**Table 1:** Sociodemographic, perinatal, and anthropometric characteristics of healthy children in the municipality of Cuiabá, Mato Grosso, Brazil

Sex	Female	869 (50.1)
(n = 1,735)	Male	866 (49.9)
Color	Yellow	51 (3.0)
(n = 1,725)	White	343 (19.9)
	Black	215 (12.5)
	Brown	1116 (64.7)
Mode of delivery	Cesarean	814 (47.1)
(n = 1,729)	Vaginal	915 (52.9)
Breastfeeding	No	293 (17.2)
(n = 1,702)	Yes	1409 (82.8)
Birth weight category (kg)	Low weight	144 (9.5)
(n = 1,511)	Normal	1270 (84.0)
	Macrosomia	97 (6.4)
	**Mean (standard deviation)**	
Birth weight (grams) (n = 1,512)		3226 (577)
Age (years) (n = 1,735)		5.3 (2.7)
Weight (kg) (n = 1,732)		20.8 (9.3)
Height (cm) (n = 1,731)		110.7 (19.0)

The variation in n results from the lack of data on some children regarding the respective variable.

**Figure 1:**
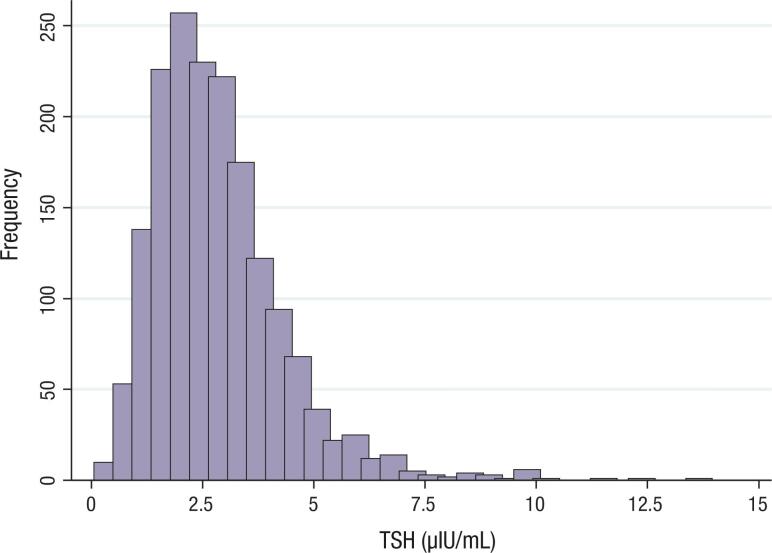
Histogram of TSH serum concentrations of children aged from 1 to 10 years of both sexes from Cuiabá (MT), 2012.

**Figure 2:**
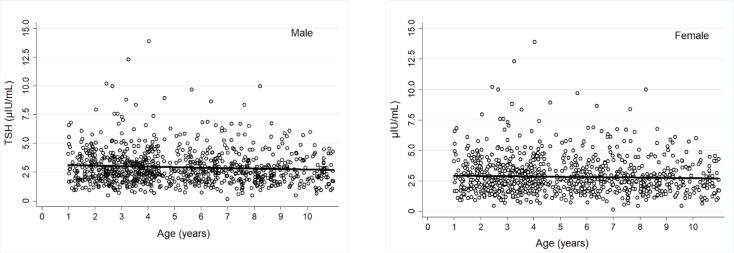
Scatter plot of TSH serum concentrations of healthy children from Central Brazil by age and sex.

The mean ± SD serum TSH concentration of all study children was 2.89 ± 1.47 μIU/mL. The distribution of serum TSH concentrations was slightly asymmetric and did not meet normality criteria (Figure 1). To identify groups of children with homogeneous serum concentrations of this hormone, the serum TSH variance was compared between boys and girls and between the 10 age groups, with a one-year interval between them. Homogeneous variances were observed between both sexes (p = 0.395) and the 10 age groups (p = 0.081) (Figure 2). For this reason, additional steps were taken to determine the serum TSH concentration RIs jointly for both sexes and for the entire group of children evaluated in this study, that is, children aged from 1 to 10 years.

**Figure 3:**
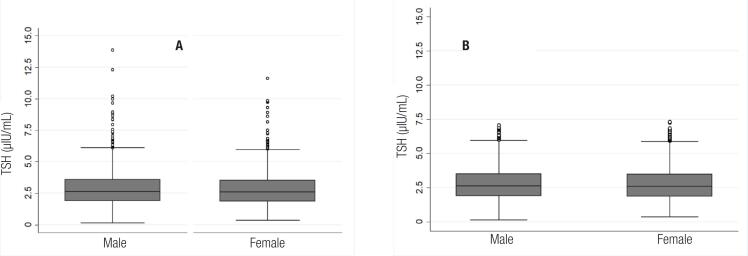
Boxplot of TSH values of boys and girls, before (A) and after (B) excluding outliers (p= 0.395)

**Figure 4:**
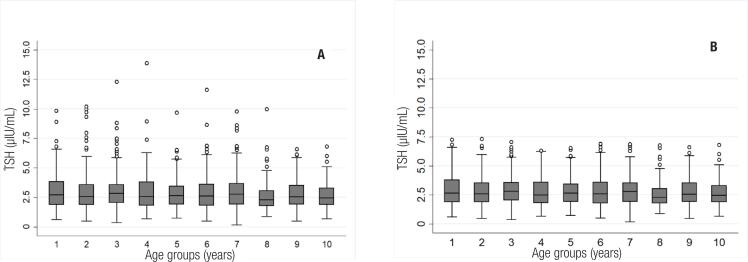
Boxplot of TSH serum concentration of boys and girls, before (A) and after (B) excluding outliers, by age group (p = 0.081).

Once TSH outliers were identified, 25 (1.4%) children were excluded from the final analysis of the RIs for this reason. The final distribution of the serum TSH concentrations of boys and girls at different ages, before and after excluding outliers, is shown in Figures 3 and 4. As shown in the floating bar charts, the serum TSH concentrations are indeed homogeneous between sexes and the 10 age groups of children aged from 1 to 10 years.

Once the outliers were removed, serum TSH concentration RIs were determined using the 2.5 and 97.5 percentile (0.93-5.86 μIU/mL) and mean ± 2 standard deviation (0.30-5.29 μIU/mL) methods. These TSH RIs of healthy Brazilian children were compared with those reported in international studies, as outlined in Table 2. Although the differences observed in the RIs compared are not considerable, both the lower limit and upper limits of the RIs show variability. This difference was observed even when breaking down the analysis by children with a similar background, by medical device from the same manufacturer and by age group of the children.

**Table 2:** Comparison of serum TSH reference intervals assessed in this study with those of international studies that used the same analytical method, grouped by homogeneity in device, data source or children's age

Author (Year)	Country	n	Data source	Age (years)	Device	RI (μIU/mL)
This study	Brazil	1,735	School	1-10	Cobas 6000 (Roche)	0.30-5.29* 0.93-5.86**
**Homogeneity in device**						
Ladang and cols. (2017)	France	1,145	Laboratory	1-13	Cobas 6000 (Roche)	0.70-5.40
Bohn and cols. (2019)	Canada	678	School	1-15	Cobas 8000 e602 (Roche)	1.12-5.01
**Homogeneity in data source**						
Aldrimer and cols. (2012)	Sweden	457	School	0.5-12	Arquitect ci8200 (Abbott)	0.89-4.97
Bohn and cols. (2019)	Canada	678	School	1-15	Cobas 8000 e602 (Roche)	1.12-5.01
**Homogeneity in children's age**						
Hübner and cols. (2002)	Germany	460	Hospital/Laboratory	1-10	Advia Centaur (Bayer)	0.42-4.79
Kapelari and cols. (2008)	Austria	1,209	Hospital	1-10	Advia Centaur (Bayer)	0.80-6.26
Soldin and cols. (2010)	USA	2,195	Outpatient center/Hospital	1-10	Arquitect ci2800 (Abbott)	0.81-4.79
Kahapola-Arachchige and cols. (2012)	Australia	820	Outpatient center/Hospital	1-10	Advia Centaur (Bayer)	0.69-3.97
Strich and cols. (2012)	Israel	6,313	Outpatient center	1-10	Advia Centaur (Bayer)	0.75-6.57

RI: reference interval; USA: United States of America.

* RI assessed using the homogeneity of variance method.

** RI assessed using the 2.5 and 97.5 percentile method.

## DISCUSSION

In the present study, the serum TSH concentration RIs of health children aged from 1 to 10 years of both sexes, living in the Central Region of Brazil were determined. The serum TSH concentration assessed in this study ranged from 0.30 to 5.29 μIU/mL based on the homogeneity of variance method and from 0.93 to 5.86 μIU/mL based on the 2.5 and 97.5 percentile method. This is the first Brazilian population-based study conducted to determine the normal serum TSH concentrations in a healthy pediatric population. Some Brazilian authors have already published studies that address the serum TSH RI in adult population (35,36). In pediatric population, the only similar study available in the literature was performed in the South Region of the country, with a convenience sample of only 71 healthy children, aged between 3 and 8 years (37). Comparing the serum TSH concentrations determined in this study with those reported in studies with large samples of the child population worldwide (5,22,27,38–42) was a challenging task because such studies used heterogeneous data sources (serum sample origin), serum TSH concentration determination methods, analytical devices and child populations.

In general, the reference values of TSH reports issued by Brazilian laboratories are defined by the Thyroid Department of SBEM (19), which, in turn, recommends that serum TSH concentrations be assessed by age and cites, as a reference, a German study published by Verburg and cols. (2011) (18), which sets a RI from 0.38 to 5.24 μIU/mL for children aged from 1 to 12 years. This study, however, was performed with a small sample of children admitted to hospitals, which represents a major selection bias (27) because these children are more likely to have elevated concentrations of this hormone due to hospitalization (12).

In a previous publication, Vieira (2010) discussed the difficulty of determining TSH RI by routine methods comparable to reference methods. In addition, inter and intra assay variations of serum TSH represent another difficulty in determining what is a normal population for this analyte (34,35). The challenge of comparing TSH RIs established in different studies also derives from the diverse data sources, ranging from electronic databases of the medical device industry to samples kept in laboratory biorepositories, and even including hospitalized or outpatient children (15,16). In addition, in determining the RI of any laboratory parameter, small variations may occur in the results when using different devices or methods (43) and, especially, different age groups of the children under study (41).

In fact, it was difficult to compile similar studies to more adequately compare the results of this study, that is, those that evaluated children in the same age groups, that had the TSH serum concentration established by the same methods and platforms, and that obtained primary population data asymptomatic infantile, as illustrated in Table 2. Such differences unquestionably have a potential impact on the resulting RIs. For example, the RIs of this study do not match those found by Aldrimer and cols. (2012) (40), who used another device to determine the serum TSH concentration. To overcome this difficulty, we compared studies with at least some similar variables.

Even using the strategy of selecting more homogeneous studies (40,42), the TSH RIs showed a variable amplitude, mainly in their upper limits. The study that used the same device (Cobas® 8000 and 602) and a sample with a similar origin (42) identified RIs similar to those found in the French study conducted by Ladang and cols. (2017) (27) but different from those found in the present study. Differences in age range (27,42) and sample origin or data source (27) stand out among the possible explanations for discrepancies between RIs. The analysis of the studies that used the same age groups as those of the children studied here also showed variability in the RIs, most likely due to other methodological heterogeneities, such as sample origin or data source and device used to determine serum TSH concentrations (5,22,38,39,41).

The TSH homogeneity between sexes observed in this research is in line with most studies conducted with children (16,17,39,42). Conversely, the TSH homogeneity observed in the group of children aged from 1 to 10 years is not found in studies that also evaluated children in the same age range (22,38,39,41). However, as shown in a specific study with a large sample of participants aged between one and over 90 years, age has a weak effect on the RI limits of serum TSH concentrations, except for elderly individuals (22).

Disregarding issues related to race and cultural characteristics of a population can increase the risk of patients in the clinical decision-making process. Even when considering a highly heterogeneous population, the interpretation of laboratory results must consider all characteristics related to the location of a given population (44). Several studies have demonstrated that ethnic and cultural differences can affect the normal reference levels of several analytes (45). A recent systematic review of racial effects on health and disease biomarkers highlighted several studies that reported differences in analyte concentration (including TSH) between ethnic groups of adults and children (46). The population of children studied here considers this factor and ensures that the serum TSH concentration RI found in this study can be extrapolated to other children in the region and even the country. The study sample originates from an area with intense migration from other regions of the country, whose racial miscegenation resulted in the population growth of Cuiabá observed at the turn of the millennium (24). Thus, despite being regional, the study population is potentially more similar to the Brazilian population than those used to establish TSH RIs in international studies.

TSH values are important in several pediatric contexts, particularly in the diagnosis and management of SH. This clinical condition is diagnosed based on limits of serum TSH concentration. SH has been implicated as a risk factor in overt thyroid dysfunction and in numerous other clinical disorders (14). SH is diagnosed by analyzing serum TSH concentration, but there is no consensus on the most appropriate RI limits. Thus, the diagnosis of this condition requires defining a normal RI first, especially for the child population.

The definition of SH varies widely between different studies, with some restricting the definition to a slightly elevated TSH concentration, for example above 4.5 mU/L, associated with a normal, age-appropriate concentration of total or free T4, whereas others use a stricter definition, with a TSH above 5.0 μIU/mL (12,13). The Brazilian consensus for the clinical approach and treatment of subclinical hypothyroidism in adults defines SH as a serum TSH concentration ≥ 4.5 mU/L, with normal concentrations of free T4, excluding other causes of elevated TSH (19), albeit without defining pediatric values, possibly due to the lack of studies in this age range. Thus, accurate TSH RI limits must be defined because misdiagnosing normal individuals with HS increases the possibility of unnecessary treatment and follow-up visits and examinations.

Considering the importance of TSH in clinical practice as a preferable marker for diagnosing thyroid diseases, the RIs of this hormone described here can be safely used in the age range from 1 to 10 years, especially when using the Cobas modular analyzer. New studies must be conducted in different regions of Brazil to determine the actual serum TSH concentration RI of the Brazilian child population. Moreover, such studies should be performed with primary data from healthy children from the community. The different immunoassay methods used to measure TSH across the country will probably not limit the comparison of the results of these studies. Different platforms for measuring TSH have shown harmonic results and excellent performance quality, as demonstrated by the IFCC Working Group on Standardization of Thyroid Function Tests (47).

The serum TSH concentration RIs of children aged from 1 to 10 years living in the Central Region of Brazil showed no sex or age differences and ranged from 0.30 to 5.29 μIU/mL when assessed using the mean ± 2 SD method and from 0.93 to 5.86 μIU/mL when assessed using the percentile method. The findings show that the serum TSH concentrations of the Brazilian children evaluated in this study differed from those found in child populations from other countries. Results from other regional population studies may corroborate the RIs found here and enable their safer use in pediatric clinical practice.
